# Top 100 Most-Cited Articles on Enhanced Recovery After Surgery: A Bibliometric Analysis and Visualized Study

**DOI:** 10.3389/fsurg.2022.845946

**Published:** 2022-05-04

**Authors:** Liping He, Lulu Lu, Shengjie Su, Qiang Lin, Chan Sheng

**Affiliations:** ^1^ Department of Nursing, The 1st People's Hospital of Baiyin, The Third Affiliated Hospital of Gansu University of Chinese Medicine, Baiyin, China; ^2^ Department of Thoracic Surgery, The 1st People's Hospital of Baiyin, The Third Affiliated Hospital of Gansu University of Chinese Medicine, Baiyin, China; ^3^ First Department of Orthopaedics and Traumatology, Baoji Traditional Chinese Medicine Hospital, Baoji, China; ^4^ Department of Emergency, The 1st People's Hospital of Baiyin, The Third Affiliated Hospital of Gansu University of Chinese Medicine, Baiyin, China

**Keywords:** ERAS, fast track, top-cited, bibliometric, visualized study

## Abstract

**Background:**

The enhanced recovery after surgery (ERAS) protocol is widely implemented in surgeries, and this study aims to reveal the characteristics of the 100 most-cited original articles in the field of ERAS research.

**Methods:**

The literature was retrieved in the Web of Science database, the 100 most-cited original articles were identified, and their characteristics were analyzed, including the trends of publications and citations; contributions from countries, institutions, and authors; co-cited authors and journals in the references; served surgeries, research endpoints, keywords; and the level of evidence.

**Results:**

There was a rising trend in the yearly publications and citations. Denmark and the USA contributed the largest number of highly cited papers. The University of Copenhagen was the most influential institution. Kehlet, Henrik was the most influential author. The *British Journal of Surgery* was the most often published and cited journal. ERAS protocols were overwhelmingly implemented in colorectal surgeries. The most focused endpoints were “length of stay”, “complications”, and “readmission”. The most frequently used keywords were “fast track”, “length of stay”, and “laparoscopy”. The keyword “enhanced recovery after surgery” burst since 2012. More than half of the highly cited articles presented level IV evidence, but there was no correlation between citations (densities) and the levels of evidence.

**Conclusions:**

The highly cited research overwhelming implemented ERAS in colorectal surgeries, the “length of stay” was the most focused element, and Kehlet, Henrik was the most influential researcher. Most of the highly cited ERAS had low levels of evidence, and the total number of citations was not relevant to the level of evidence. Therefore, studies with high levels of evidence are still required in the future.

## Introduction

In 2001, researchers from five countries gathered in Europe and formed the Enhanced recovery after surgery (ERAS) study group (1). This group aimed to promote the implementation of ERAS protocols and has produced several guidelines (2–5). Before this period, “fast-track surgery” had been practiced for several years. Engelman et al. first reported fast-track recovery for coronary artery bypass surgery in 1994 (6), followed by research from Bardram et al. and Kehlet et al. (7, 8). Although the ERAS study group wanted to emphasize that quality rather than the speed of rehabilitation should be focused (1), the concept “fast-track” has yet to be widely used. Besides, fast-track anesthesia included not only early extubation but also multimodal pain management perioperatively, and the development of fast-track anesthesia and analgesia was greatly promoted by ERAS research (7). It is necessary to reveal the highly cited articles during the evolution of ERAS research. The bibliometric analysis could evaluate the scientific value of the literature quantitatively (9–11), and this study identified the 100 most-cited original ERAS research articles and analyzed their characteristics, which represent the most influential papers in this field.

## Methods

### Literature Search and Screening

The Web of Science (WOS) database was searched on April 2021 by using the following strategy: “TS = enhanced recovery after surgery” OR “fast-track surgery” OR “fast-track rehabilitation” OR “fast-tracking rehabilitation” OR “multimodal rehabilitation”. The year was set from 1990 to 2020, without any language restrictions.

Articles were screened on the WOS website online according to the following protocols: Only original articles were included, reviews, meta-analyses, guidelines, case reports, letters, editorial materials, meeting abstracts, expert experiences, trial protocols, animal studies, and articles that did not discuss ERAS were excluded. The results were ranked by citations, and articles that meet the selection criteria were added to the “marked list” until there were 100 items. Two authors screened the articles independently, and the final agreement was achieved after discussion.

### Publications and Citations

Articles were imported into literature management software Endnote (version X9, Clarivate Analytics, Philadelphia, PA, USA) and Microsoft Excel (version 2019, Microsoft Corp. Redmond, WA, USA) for analysis. The number of yearly publications and citations was counted. The distribution of institutions and countries was determined by corresponding authors. The surgery names and endpoints were distracted from the articles by looking through the full texts. The level of evidence was graded by using the Oxford Centre for Evidence-Based Medicine (OCEM) 2011 grading system (12), two authors graded the levels independently, and the agreement was calculated by kappa consistency analysis; the discrepancy was solved by discussing with the third author. The citations and citation densities (total citations/the number of years since published) were compared between different levels of evidence.

### Visualized Analysis

Vosviewer (version 1.6.16, Leiden University, Leiden, The Netherlands) was used for visualized analysis, including bibliographic coupling analysis of authors and journals, co-cited analysis of cited authors and journals, and co-occurrence analysis of keywords and endpoints. Visualized networks were made and the total link strengths between individuals were recorded. The burst of keywords was detected by using Citespace (version 5.7, Drexel University, Philadelphia, PA, USA), and keywords that frequently occurred during certain periods were shown in the burst map.

### Statistical Analysis

All statistical analyses were completed by SPSS (version 25.0, IBM Corporation, Armonk, NY, USA). The Kolmogorov–Smirnov test was used to determine whether the distribution of continuous variables was normal, and non-normally distributed data were presented as the median (interquartile range, *IQR*). A comparison between multiple variables was tested by using the Kruskal–Wallis H-test. The correlation between the two variables was tested by using the Spearman test. Consistency between two independent authors during the grading of the levels of evidence was determined by using the Kappa consistency test. A value of *p *< 0.05 was considered significantly different.

## Results

### Publications and Citations

**Figure 1:**
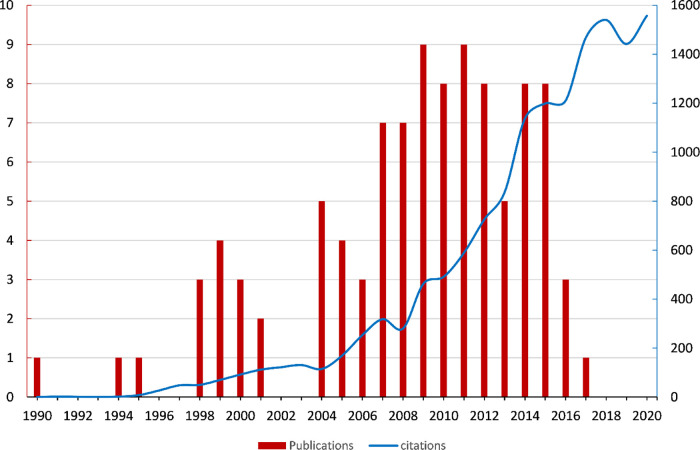
Trends of yearly publications from 1990 to 2016, and yearly citations from 1990 to 2020.

All of the top 100 cited articles were from WOS Core Collection and were written in English. The citations ranged from 80 to 547 times by the time we retrieved them, with a median citation of 108 (90.25, 103.75) and an h-index of 85. The total number of citations was 14,456 (14,203 without self-citations). The top 100 most-cited articles were published from the years 1990 to 2016. The citation data were analyzed from 1990 to 2020. There was a rising trend in the number of publications and citations (**Figure 1**), and the Spearman test showed a strong correlation between years and publications (*ρ* = 0.986, *p *< 0.001); years and citations (*ρ* = 0.752, *p *< 0.001).

### Countries and Institutions

**Figure 2:**
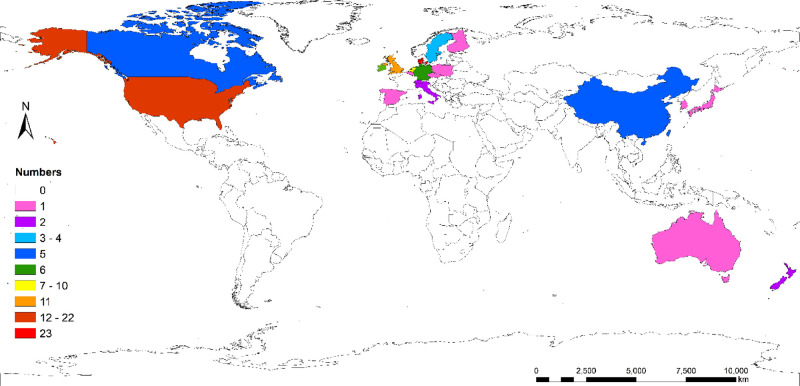
World distribution of top 100 cited articles.

The top 100 cited articles were from 19 countries and 58 institutions. Denmark contributed the greatest number of publications and citations, with 23 articles and 4,099 citations, followed by the USA, with 22 articles and 2,848 citations. Netherlands and the UK each contributed 10 articles (**Figure 2**). The top 10 countries with the greatest number of publications are listed in **Table 1**. Hvidovre Univ Hosp owned the largest publications and citations, with 16 articles and 3,098 citations, followed by Rigshosp and St Marks Hosp. The top 10 institutions with the greatest number of publications are listed in **Table 2**.

**Table 1:** Top 10 countries with the greatest number of publications.

Country	Publications	Citations	Citations per item
Denmark	23	4,099	178.2
USA	22	2,848	129.5
Netherlands	10	1,777	177.7
UK	10	1,518	151.8
Germany	6	647	107.8
Canada	5	488	97.6
China	5	544	108.8
Sweden	4	800	200
Italy	2	282	141
New Zealand	2	230	115

**Table 2:** Top 10 corresponding institutions with the greatest number of publications.

Institution	Country	Publications	Citations
Hvidovre Univ Hosp	Denmark	16	3,098
Rigshosp	Denmark	5	743
St Marks Hosp	UK	4	816
Ersta Hosp	Sweden	4	800
Univ Texas	USA	4	501
Acad Med Ctr	Netherlands	3	715
Cleveland Clin Fdn	USA	3	452
Univ Hosp Maastricht	Netherlands	2	415
Univ Hosp CHUV	Switzerland	2	351
Univ Virginia Hlth Syst	USA	2	284

### Authors

**Figure 3:**
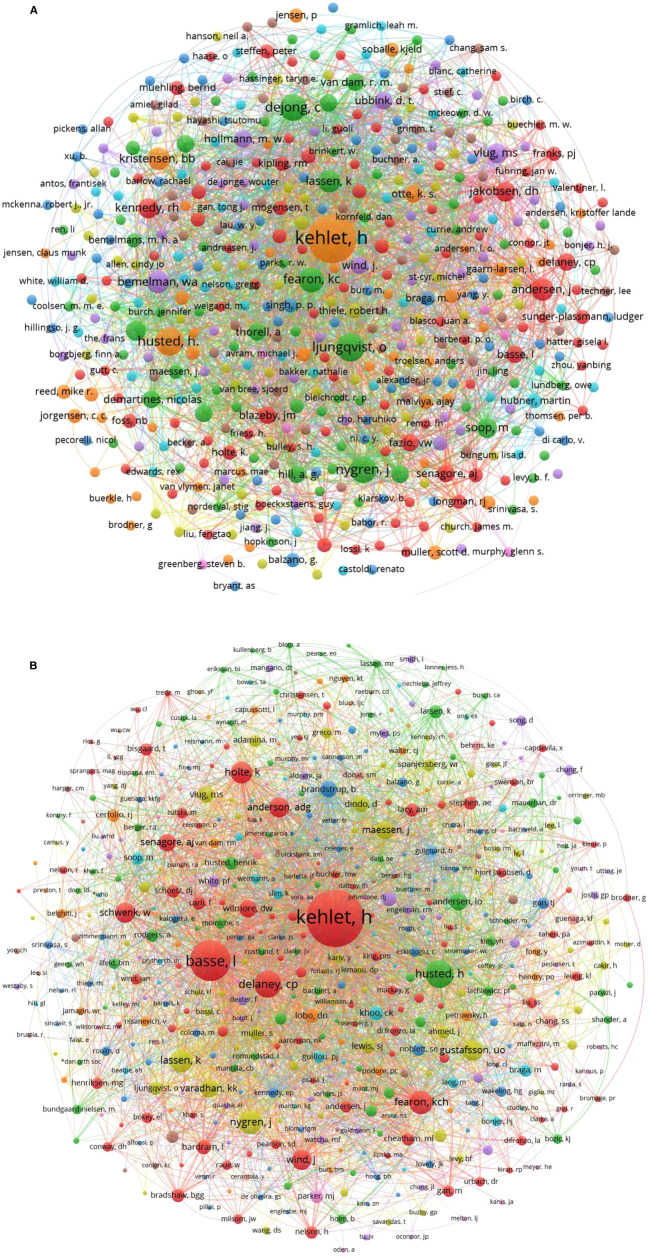
(**A**) Bibliographic coupling analysis of authors; (**B**) Co-cited analysis of cited authors in the references.

Kehlet, Henrik was the author with the greatest number of publications and citations, with 22 articles and 3,713 citations, followed by Ljungqvist, Olle, and Husted, Henrik, with each of them contributing 8 articles and having a citation of 1,530 and 1,102, respectively (**Figure 3A**). The top 10 authors with the largest contributions are listed in **Table 3**. In terms of cited authors in the reference lists, Kehlet, Henrik was cited 141 times, and the total link strength was 3,550, followed by Basse, Line Hollesen, who was cited 73 times (**Figure 3B**). The top 10 cited authors in the reference lists are listed in **Table 4**.

**Table 3:** Top 10 authors with the greatest number of publications.

Author	Affiliation in articles	Country	Publications	Citations
Kehlet, Henrik	Rigshospitalet	Denmark	22	3,713
Ljungqvist, Olle	Orebro University	Sweden	8	1,530
Husted, Henrik	Rigshospitalet	Denmark	8	1,102
Nygren, Jonas	Ersta Sjukhus	Sweden	7	1,450
Dejong, Cornelis HC	Maastricht University	Netherlands	7	906
Fearon, Kenneth CH	University of Edinburgh	UK	6	1,032
Lassen, Kristoffer	National Hospital Norway	Norway	5	960
Bemelman, Willem A	University of Amsterdam	Netherlands	5	934
Andreasen, Jens Ove	Rigshospitalet	Denmark	5	736
Kristensen, Billy B	Abt Ambulante Chirurg	Denmark	5	596

**Table 4:** Top 10 most-cited authors in the reference lists.

Author	Affiliation in articles	Country	Cited times	Total link strength
Kehlet, Henrik	Rigshospitalet	Denmark	141	3,550
Basse, Line Hollesen	Novo Nordisk	Denmark	73	1,091
Husted, Henrik	University of Copenhagen	Denmark	34	808
Delaney, Conor P	Dana-Farber Cancer Institute	USA	31	811
Fearon, Kenneth CH	University of Edinburgh	UK	25	676
Nygren, Jonas	Karolinska Institutet	Sweden	24	718
Lassen, Kristoffer	National Hospital Norway	Norway	23	620
Holte, Kathrine	Rigshospitalet	Denmark	21	600
Gustafsson, Ulf O	Danderyds Hospital	Sweden	19	539
Wind, Jan	University of Amsterdam	Netherlands	18	508

### Journals

The top 100 cited articles were published in 33 journals. The most often published journal was the *British Journal of Surgery*, with 14 articles and 2,200 citations, followed by *Annals of Surgery* and *World Journal of Surgery*; the top 10 most often published journals are listed in **Table 5**. The *British Journal of Surgery* was the most-cited journal in the references, followed by *Annals of Surgery*, *Anesthesia and Analgesia*. Spearman correlation test showed a weak correlation between impact factors and citations (*ρ* = 0.243, *p* = 0.015).

**Table 5:** Top 10 most often published journals.

Journal	Publications	Citations	Citations per item	IF (2020)
*British Journal of Surgery*	14	2,200	157	6.939
*Annals of Surgery*	7	1,967	281	12.969
*World Journal of Surgery*	7	677	97	3.352
*Anesthesia and Analgesia*	6	765	128	5.178
*Acta Orthopaedica*	5	886	177	3.717
*Anesthesiology*	5	456	91	7.892
*Journal of Gastrointestinal Surgery*	4	365	91	3.452
*Colorectal Disease*	4	396	99	3.788
*Diseases of the Colon & Rectum*	3	452	151	4.785
*Clinical Nutrition*	3	266	89	7.325

### Surgeries and Study Endpoints

**Figure 4:**
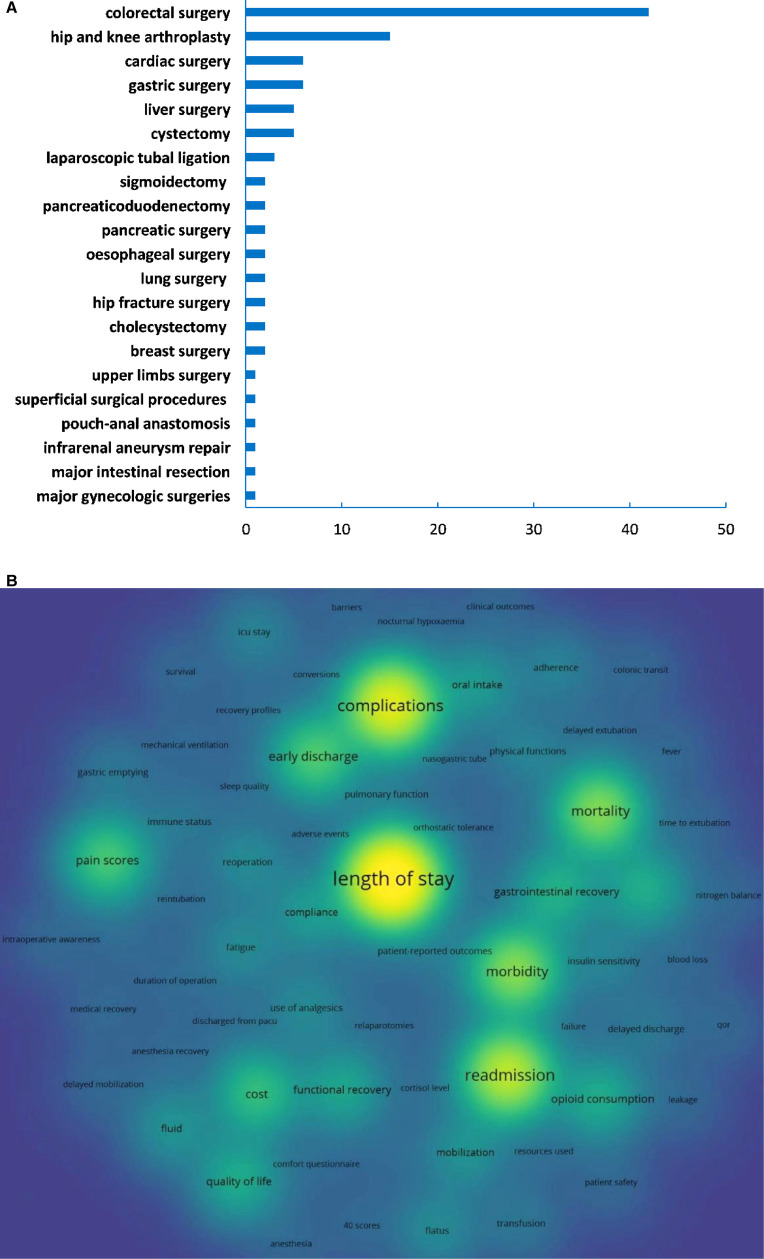
(**A**) Distribution of surgeries; (**B**) Co-occurrence analysis of study endpoints.

Most of the top 100 cited articles investigated the implementation of ERAS protocols in colorectal surgeries, followed by hip and knee arthroplasties, cardiac surgery, and gastric surgery (**Figure 4A**). The most focused endpoints were the length of stay, followed by complications, readmissions, morbidities, and mortalities (**Figure 4B**). Among them, 27 articles mentioned the primary endpoints, and 14 of them were about the length of stay (51.85%).

**Figure 5:**
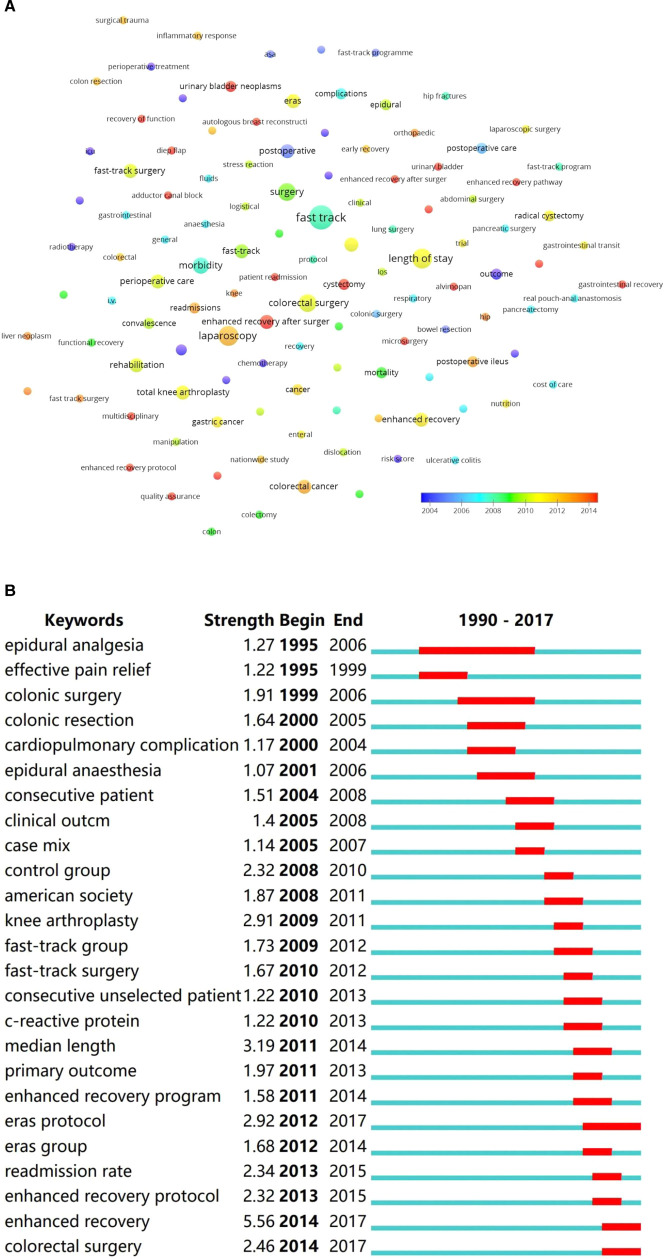
(**A**) Co-occurrence analysis of author keywords, ranging by year; (**B**) Burst detection of author keywords from 1990 to 2020.

**Keywords:** The most often occurred keywords were “fast track”, “length of stay”, “laparoscopy”, “surgery”, “colorectal surgery”, and “morbidity” (**Figure 5A**). The burst of keywords showed that “epidural analgesia”, “colonic surgery”, and “fast-track” were keywords that burst in the early years of ERAS research, while the “enhanced recovery” burst since 2012 (**Figure 5B**).

### Study Design and Level of Evidence

**Figure 6:**
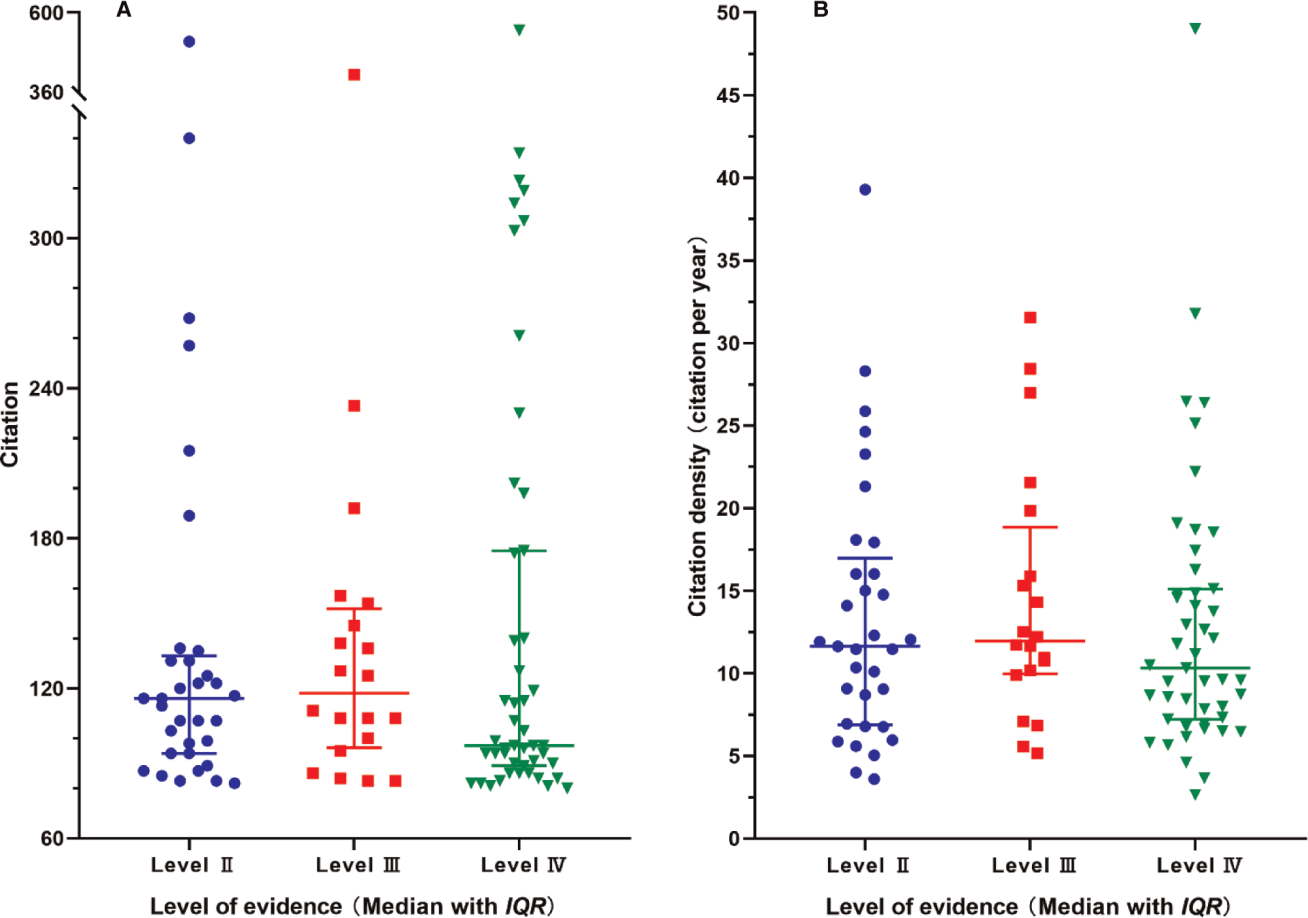
Distribution of citations (**A**) and citation densities (**B**) between different levels of evidence.

There were 33 articles with level II evidence, 20 articles with level III evidence, and 57 articles with level IV evidence. There was no significant difference in citations (*Z* = 1.077, *p* = 0.584) (**Figure 6A**) and citation densities (*Z* = 1.227, *p* = 0.541) between different levels of evidence (**Figure 6B**). The most often used study designs were randomized controlled trials (RCTs), retrospective case serious, prospective case serious retrospective, and case-control studies. The study designs of the top 100 cited articles are listed in **Table 6**.

**Table 6:** Study designs of the top 100 cited articles.

Design	*n*
Prospective, case serious	13
Prospective, non-randomized controlled cohort	3
Prospective, non-randomized controlled follow-up	9
RCT	33
Retrospective, case serious	19
Retrospective, case-control study	11
Retrospective, non-randomized controlled cohort	6
Combined, non-randomized controlled cohort	2
Combined, case-control study	1
Others	3

## Discussion

The citation analysis is the most widely used tool in evaluating the academic impact (13–21). The present study identified the 100 most-cited original ERAS research and analyzed their characteristics, which will help researchers quickly find the most influential contributors and gather research interests. We retrieved literature and confirmed that this is the first bibliometric analysis of the top 100 most-cited articles in ERAS research.

Kehlet, H, a member of the first ERAS study group, was the most influential author in the field of ERAS research. We found that many influential institutions belong to the University of Copenhagen. The top three most frequently cited authors in the references were all from Denmark. In this study, the contribution of authors and institutions was not equal because the former was calculated by a full-counting bibliographic coupling analysis, while the latter was determined by corresponding authors. The corresponding author was considered the major contributor to an article; however, this may lead to bias. Another phenomenon was that articles from inventors or pioneers were frequently cited, and, thus, the distribution of institutions and countries can be greatly influenced by some productive authors. So, an evaluation of influential contributors should be based on comprehensive criteria. The distribution of the highly cited articles reflects the unbalanced implementation and compliance of ERAS protocol, as well as the academic influence of the contributors.

The top-cited article performed a multimodal rehabilitation program of 48-hour postoperative stay for patients undergoing colonic resection (22). The second most-cited article was a multicenter, randomized clinical trial that compared the laparoscopic and open resection of colon cancer combined with fast-track care (23). The third most-cited article found that improved adherence to ERAS protocol significantly improved the outcomes of patients undergoing colorectal surgery (24). It was not feasible to summarize all of the top-cited articles here, however, underscoring that major topics of the most highly cited articles are necessary. To better guide clinical practice, we retrieved and listed the top 10 most-cited guidelines in the ERAS field (**Table 7**), though they were not included in this bibliometric study. We found that the top 10 cited guidelines were almost from the Enhanced Recovery After Surgery (ERAS) Society. When analyzed by the record counts of authors, Gustafsson, UO was the most frequently cited author, and the top cited affiliations were similar to those of the top 100 cited original articles. The top 10 most-cited guidelines were compiled for the clinical practices of elective colonic surgery (25), elective colorectal surgery (26), radical cystectomy for bladder cancer (27), gastrectomy (28), lung surgery (29), elective rectal/pelvic surgery (30), pancreaticoduodenectomy (31), liver surgery (32), bariatric surgery (33), and elective rectal/pelvic surgery (34), respectively. Colorectal surgery also owns the overwhelming number of citations in ERAS guidelines. It was worth noting that orthopedic surgery lacks highly cited guidelines, though it ranks second in the total citations of original research.

**Table 7:** Top 10 most-cited ERAS guidelines.

Author	Year	Title	Journal	Cited times
Gustafsson, UO et al.	2012	Guidelines for perioperative care in elective colonic surgery: Enhanced Recovery After Surgery (ERAS (R)) society recommendations	*Clinical Nutrition*	605
Gustafsson, UO et al.	2019	Guidelines for perioperative care in elective colorectal surgery: Enhanced Recovery After Surgery (ERAS(R)) society recommendations: 2018	*World Journal of Surgery*	501
Cerantola, Y et al.	2013	Guidelines for perioperative care after radical cystectomy for bladder cancer: Enhanced Recovery After Surgery (ERAS(R)) society recommendations	*Clinical Nutrition*	349
Mortensen, K et al.	2014	Consensus guidelines for enhanced recovery after gastrectomy Enhanced Recovery After Surgery (ERAS(R)) society recommendations	*British Journal of Surgery*	297
Batchelor, TJP et al.	2019	Guidelines for enhanced recovery after lung surgery: recommendations of the Enhanced Recovery After Surgery (ERAS((R))) society and the European Society of Thoracic Surgeons (ESTS)	*European Journal of Cardio-Thoracic Surgery*	281
Nygren, J et al.	2013	Guidelines for perioperative care in elective rectal/pelvic surgery: Enhanced Recovery After Surgery (ERAS(R)) society recommendations	*World Journal of Surgery*	268
Lassen, K et al.	2012	Guidelines for perioperative care for pancreaticoduodenectomy: Enhanced Recovery After Surgery (ERAS(R)) society recommendations	*Clinical Nutrition*	261
Melloul, E et al.	2016	Guidelines for perioperative care for liver surgery: Enhanced Recovery After Surgery (ERAS) society recommendations	*World Journal of Surgery*	246
Thorell, A et al.	2016	Guidelines for perioperative care in bariatric surgery: Enhanced Recovery After Surgery (ERAS) society recommendations	*World Journal of Surgery*	241
Nygren, J et al.	2012	Guidelines for perioperative care in elective rectal/pelvic surgery: Enhanced Recovery After Surgery (ERAS(R)) society recommendations	*Clinical Nutrition*	232

The most often published journals reflect their high reputations within the ERAS research. The co-citation analysis revealed the most high-impact journals. Revealing the highly cited journals can also help researchers quickly locate authoritative publishers, as well as providing a reference when they submit manuscripts. By reading high-quality research, it is easy for readers to quickly locate these authoritative contributors and to learn what these highly cited papers focus on. However, the major limitation is that this bibliometric analysis did not include reviews, which summarize the newest research trends and are highly cited. This bibliometric analysis just focused on original clinical research and aimed to provide more guidance for clinical practice. As was mentioned above, the impact factor had several limitations in measuring scientific values. We found that it was not significantly correlated to the citations even in highly cited articles. The academic values of journals should also be comprehensively evaluated.

The ERAS protocol was overwhelmingly implemented in colorectal surgeries, followed by hip and knee arthroplasties. The disruption of gastrointestinal functions, susceptibility to surgical trauma in the elderly, and physical weakness in malignancies were strong indications for ERAS protocols (35–37). The efficacy of ERAS was more often measured by the length of stay, complications, and readmission rate. Among the articles that mentioned primary endpoints, half of them pertained to the length of stay, suggesting that it was the most focused outcome in ERAS research. The co-occurrence analysis of keywords was weighed by the average occurred years, and it presented similar results to that of burst detection. Although the ERAS concept was put forward in 2000, “fast-track” was still the most often used keyword and burst in the early years of ERAS research. The keyword “enhanced recovery after surgery” burst since 2012, and it ended in 2017 because there were no top-cited articles in the recent 3 years. Top-cited articles can reflect research interests and trends in some ways, though their representativeness is limited.

According to the OCEM 2011 level of evidence system (12), RCT counted one-third among all study designs, and there was no level I evidence because systemic reviews and meta-analyses were not included for analysis. More than half of the highly cited articles were observational studies and presented level IV evidence. Interestingly, there was no significant difference in total citations and citation densities between different levels of evidence, indicating that even articles with low levels of evidence can be cited many times. Although well-designed observational studies are easier to be implemented and can be widely cited, articles with high levels of evidence are still required.

The present study has several limitations. First, the literature was retrieved in the WOS database, and highly cited articles from other databases may be dismissed. Furthermore, the counting of citations can differ between databases. Second, the distributions of countries and institutions were determined by the corresponding authors, while the authors’ contributions were analyzed by full-counting analysis, and this may lead to bias. Third, the list did not contain articles published in the recent 3 years because the citations can be influenced by article age, and some newly published articles had a limited number of citations despite their high-impact values. Despite these facts, this is the first bibliometric analysis of top-cited articles in the field of ERAS research and clearly showed the major contributors and research interests.

## Conclusions

Revealing highly cited articles can help researchers quickly find important contributions and gather research interests. The highly cited research overwhelmingly implemented ERAS in colorectal surgeries, the “length of stay” was the most focused element, and Kehlet, Henrik was the most influential researcher. Most of the highly cited ERAS had low levels of evidence, and the total number of citations was not relevant to the level of evidence. Therefore, studies with high levels of evidence are still required in the future.

## Data Availability

The original contributions presented in the study are included in the article/Supplementary Material, and further inquiries can be directed to the corresponding author/s.
